# Cenicriviroc blocks HIV entry but does not lead to redistribution of HIV into extracellular space like maraviroc

**DOI:** 10.7448/IAS.17.4.19531

**Published:** 2014-11-02

**Authors:** Victor Kramer, Said Hassounah, Susan Colby-Germinario, Thibault Mesplède, Eric Lefebvre, Mark Wainberg

**Affiliations:** 1 McGill AIDS Centre, Lady Davis Institute, Jewish General Hospital, Montreal, QC, Canada; 2 Tobira Therapeutics, Inc., South San Francisco, CA, USA

## Abstract

**Introduction:**

Cenicriviroc (CVC), a once-daily, dual CCR5/CCR2 co-receptor antagonist, has completed Phase 2b development. CVC demonstrated favourable safety and similar efficacy compared with efavirenz (EFV) in Study 202 (NCT01338883); an *ex vivo* sub-analysis evaluated treatment effects on HIV entry, measured by intracellular HIV DNA declines, in subjects with virologic success at Week 24. In addition, *in vitro* assays determined and compared the extent of any cell-free virion redistribution that CVC or maraviroc (MVC) may cause.

**Methods:**

*Ex vivo*: intracellular DNA (frozen PBMCs) from 30 subjects with virologic success at Week 24 (10, 13 and 7 subjects on CVC 100 mg, CVC 200 mg and EFV, respectively). Early (strong-stop) and late (full-length) reverse transcript levels were measured by qPCR. *In vitro*: PM-1 cells were infected with CCR5-tropic HIV-1 BaL in the presence or absence of inhibitory concentrations of CVC (20 nM), MVC (50 nM) or controls. P24 and viral load levels were measured by ELISA and qRT-PCR after 4 hours.

**Results:**

*Ex vivo* analysis showed full-length HIV DNA declines were similar across all groups (CVC 100 mg, CVC 200 mg and EFV) at Week 24. Strong-stop HIV DNA declines (a marker of HIV entry) at Week 24 were pronounced for both CVC arms (CVC 100 mg, 51% decline; CVC 200 mg, 37% decline) compared to no decline for the EFV arm. *In vitro* experiments revealed that CVC-treated cells had lower levels of supernatant P24 at 4 hours versus baseline (0 hrs: 506 ng/mL; 4 hrs: 192 ng/mL), but P24 levels remained constant for MVC-treated cells after 4 hours (0 hrs: 506 ng/mL; 4 hrs: 520 ng/mL). Viral load levels for CVC-treated cells remained stable after 4 hours (0 hrs: 1.19×10^10^ copies/mL; 4 hrs: 1.26×10^10^ copies/mL). MVC-treated cells exhibited a slight increase in viral load after 4 hours (0 hrs: 1.19×10^10^ copies/mL; 4 hrs: 1.67×10^10^ copies/mL).

**Conclusions:**

*Ex vivo* analysis confirmed that CVC treatment blocks HIV entry (strong-stop HIV DNA declines), while *in vitro* analysis showed that CVC-treated cells do not repel virus back into the extracellular space, as seen with MVC. Experiments are underway to determine whether or not interactions between CVC and HIV at the binding site may explain these unanticipated findings.

